# FAC-Net: Feedback Attention Network Based on Context Encoder Network for Skin Lesion Segmentation

**DOI:** 10.3390/s21155172

**Published:** 2021-07-30

**Authors:** Yuying Dong, Liejun Wang, Shuli Cheng, Yongming Li

**Affiliations:** College of Information Science and Engineering, Xinjiang University, Urumqi 830046, China; xj_dyy@stu.xju.edu.cn (Y.D.); slcaydxju@stu.xju.edu.cn (S.C.); lym@xju.edu.cn (Y.L.)

**Keywords:** skin lesion segmentation, feedback fusion, attention mechanism, lightweight model

## Abstract

Considerable research and surveys indicate that skin lesions are an early symptom of skin cancer. Segmentation of skin lesions is still a hot research topic. Dermatological datasets in skin lesion segmentation tasks generated a large number of parameters when data augmented, limiting the application of smart assisted medicine in real life. Hence, this paper proposes an effective feedback attention network (FAC-Net). The network is equipped with the feedback fusion block (FFB) and the attention mechanism block (AMB), through the combination of these two modules, we can obtain richer and more specific feature mapping without data enhancement. Numerous experimental tests were given by us on public datasets (ISIC2018, ISBI2017, ISBI2016), and a good deal of metrics like the Jaccard index (JA) and Dice coefficient (DC) were used to evaluate the results of segmentation. On the ISIC2018 dataset, we obtained results for DC equal to 91.19% and JA equal to 83.99%, compared with the based network. The results of these two main metrics were improved by more than 1%. In addition, the metrics were also improved in the other two datasets. It can be demonstrated through experiments that without any enhancements of the datasets, our lightweight model can achieve better segmentation performance than most deep learning architectures.

## 1. Introduction

Among malignant tumors affecting the elderly worldwide, skin cancer is common; there are approximately 5.4 million skin disease cases added every year [1]. Melanoma is the most deadly [2,3]. Primary melanoma is usually confined to the skin surface cells. Melanoma cancer cells will invade other tissues and organs of the human body (such as lung and brain) through the circulatory system when the disease worsens. If patients get timely discovery and treatment, the cure rate may reach more than 95%. However, the cure rate for advanced melanoma is only 15% [4]. Therefore, a timely diagnosis of melanoma is essential.

Dermoscopy is the primary means of improving the rate of skin cancer diagnosis and decreasing skin cancer mortality [5]. This method can visualize precisely the structure of the lesion in the skin at the level of the pixels. However, in clinical medicine, we have found that manual dermatoscopic visual inspection of skin lesions consumes a great deal of doctors’ time and energy, and in the process of diagnosis, different doctors will make subjective decisions based on their own experience [6]. Lately, it is because of these problems that the technology of segmentation of the dermoscopic image was born (which is the segmentation of damaged skin from the background of the dermoscopic image).

In recent years, with the rapid development of deep learning, computer aided diagnosis (CAD) systems are developing continuously. With respect to this visual evaluation of dermatoscopy images, CAD can provide quantitative and objective results, and in clinical trials, CAD has helped dermatologists to improve their clinical diagnostic accuracy for melanoma. A standard method of skin lesion inspection currently used consists of five steps: imaging, pre-treatment, segmentation, feature extraction, and classification [7]. In the process, segmenting the lesion portion of the dermoscopic image allows the physician to focus more on the lesion area, which can improve the physicians’ diagnostic effectiveness.

Early segmentation methods for skin lesions usually use algorithms based on optimal threshold, region growing, and edge detection. Since traditional methods require manual intervention, more and more experts and scholars have begun to explore more efficient segmentation methods; various segmentation methods based on CNN have been proposed and improved. However, these existing methods still have limitations. The reasons for this are as follows: first, the information extracted from the current network is underused. Second, the network does not take into consideration the relationship between contexts when extracting features. Third, the attention mechanism does not take into full consideration the multi-scale information in the network.

Facing the above challenges, an innovative FAC-Net was proposed by us to better segment skin lesions. Specifically, we propose an FFB module for the first time and use it among the down-sampling process of the network. The FFB has three advantages: First, it can apply the output results to the input maps through feedback, to further strengthen the crucial information in the feature map, and then fully extract and use global information. Second, it can convert the original output result feature map into the weight matrix of the original input feature map after maximum global pooling, so as to achieve the effect of weighting and to emphasize the original feature map information. Third, it can fuse feature map information with different resolutions from two adjacent layers to get a feature map with more comprehensive information, and ultimately strengthen the segmentation result. Based on the three advantages mentioned above, we can see that the network proposed in this paper can get the information on skin lesions more comprehensively, to improve the accuracy of segmentation for lesions. In the up-sampling, we apply the AMB module after the skip connection. We pass the enormous amount of information gotten in this step through the AMB module to extract the critical information after enhancement and suppress irrelevant and erroneous information. We extensively evaluated the network proposed by us on three datasets (ISBI2016, ISBI2017, ISIC2018). Experimental results show that without using data augmentation, we get better segmentation results through the FAC-Net network than most deep learning networks.

We summarize the contributions of this article below:

- We proposed a novel and efficient FFB, which captures multi-scale features and fuses information on different scales to get richer information of feature maps.
- The AMB module is an improved Convolutional Block Attention Module, which applies to skip connection fusion, strengthening vital information and suppressing irrelevant interference information.

We introduce existing work in Section 2. Then, we describe the method and its corresponding analysis in Section 3. The experimental settings, details, results, and evaluation indicators are introduced in Section 4. Next, we describe the discussion of the network, draw some conclusions and look forward to the future in Section 5.

## 2. Related Work

In this part, we mainly introduce three parts, namely segmentation network, feedback mechanism, and attention mechanism.

### 2.1. Segmentation Network

Skin lesion segmentation [8] is to distinguish the diseased part and the normal part of the patients’ skin by artificial or others means, then accurately segment the diseased area, to prepare for the doctor’s further diagnosis and treatment. Traditional skin lesion segmentation algorithms include threshold-based [9], edge detection [10], region growth [11], and active contour-based [12] segmentation methods. These traditional methods still have successful applications in medical images; Dang et al. [13] proposed a color model of normalization based on adaptive thresholding and obtained better results than the Otsu segmentation method. Militello et al. [14] proposes a semi-automatic method to assist the cardiologist in gaining personalized diagnosis and therapy. As deep learning continues to develop in the field of computer vision in recent years, more and more experts and scholars are actively exploring the application of deep learning in various fields, including the segmentation of skin lesions. It achieved remarkable results in the segmentation of skin lesions. Ben-Cohen et al. [15] first explored the use of FCN to complete the segmentation task of liver and tumor in CT images. Yuan et al. [16] trained an end-to-end skin melanoma segmentation method based on 19-layer FCN. Then, experts have proposed a series of segmentation methods based on deep learning, such as SegNet [17] and Deeplab [18] based on fully convolutional neural networks, which have continuously improved the accuracy and efficiency of segmentation. Ronneberger et al. [19] proposed U-Net, and this U-Net architecture is a landmark in the field of medicine. Subsequently, a series of networks based on U-Net have appeared in the field of computer vision, such as R2U-Net [20], context encoder network (CE-Net) [21], and U-Net++ [22]. In addition, U-Net3+ [23] proposed by Huang et al. uses full-scale skip connections and deep supervision to improve the problem of insufficient information extraction. The network SA-UNet [24] adds a spatial attention mechanism on the basis of U-Net to achieve adaptive optimization. Phan et al. [25] proposed an adjustable skip connection, which solves the problem of large scale variation among layers by performing an adjustable skip connection operation through a selective kernel module. Salih et al. [26] decomposed the likelihood function, which more effectively gave play to the advantages of the combination of the pixel-based MRF model and random region. Khan et al. [27] used local color-controlled histogram intensity values (LCcHIV) to enhance the input image to enrich the information. Tong et al. [28] used a combination of three attentional mechanisms to focus the neural network on the visual field more relevant to the segmentation target. Hafhouf et al. [29] combined the extended convolution and pyramid pooling module and used it in the codec structure to improve the segmentation result. Saha et al. [30] proposed a color enhancement technique that adaptively enhances the data and distinguishes the structural features of normal skin from damaged skin tissue through deep visualization. Tang et al. [31] proposed to use context information to guide the feature coding process, and adopted a new deep monitoring objective function to supervise the entire network end-to-end. Wu et al. [32] proposed an efficient and adaptive dual-attention module. Meanwhile, the backbone network adopts a dual-coding structure, which reduces redundancy and expands the network’s reception domain.

### 2.2. Feedback Mechanism

The feedback mechanism allows the network to carry the output information to modify the state of the input. In the network, feedback mechanisms enable adequate multiplexing of parameters, reducing the introduction of other parameters. It achieves feedback propagation of feature information by incorporating features from each round of iteration into the feature input of the next round of iteration. The model incorporated into this module will have fewer parameter quantities as well as faster execution. It has been used many times in different vision tasks [33,34]. Recently, feedback mechanisms have been adopted by many network architectures to meet various computer vision tasks. At the same time, semantic segmentation [35] tries to use the topology loss to extract high-level language information, and high-level language information is fed back to the shallow network to correct low-level semantic information. Then, it converts important output information into input image information to solve the classification problem in computer vision tasks.

### 2.3. Attention Mechanism

The traditional CNN network ignores the dependencies between feature maps when extracting features, such as the dependency between space and channel. Based on this problem, more and more experts and scholars have explored better mechanisms to establish connections between feature maps. Bahdanau et al. [36] first consider the relationship between gained features to improve the acquisition of critical features in natural language translation. Wang et al. [37] proposed a non-local block to obtain the dependence of the global information on the pixel-level relationship. In addition, Hu et al. [38] proposed and used the squeeze excitation (SE) module to obtain the weight map, which weighted the gained feature information to emphasize vital information. Cao et al. [39] proposed that GCNet is based on non-local blocks and SE blocks, recalibrating the dependence between dimensions in the network at the pixel level. In recent years, in order to further capture the correlation of features in each dimension, a method based on the fusion of spatial attention and channel attention has been proposed (Convolutional Block Attention Module (CBAM) [40], CCNet [41] and Dual attention [42]). In addition, self-attention mechanisms have been very recently used in Generative Adversarial Networks (GANs) for unsupervised anomaly detection on MRI, like Han et al. [43] applied SA module between specified convolutional layer and batch normalization layer to realize the recalibration and transfer of effective features of the network by establishing long-term dependence among features, ignoring the interference of irrelevant information.

## 3. Methods

In this chapter, the overall framework of the following FAC-Net was introduced in brief firstly. Then, the composition, structure and implementation details of the FFB module are also introduced by us. Finally, we introduce the AMB module in detail.

### 3.1. The Overall Structure of FAC-Net

**Figure 1:**
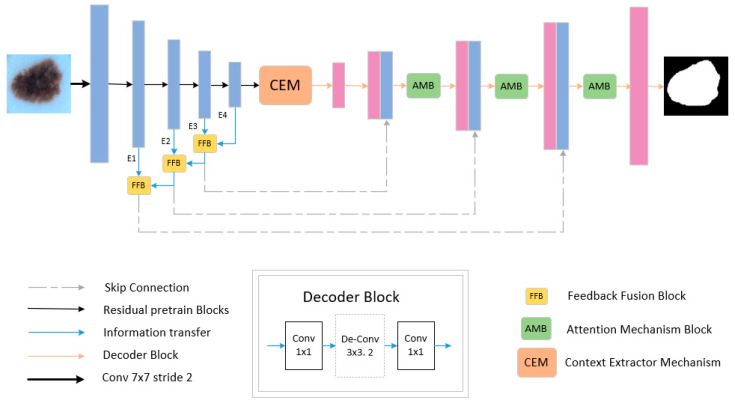
The overall design of FAC-Net. The dashed line indicates a skip connection. The yellow block represents the feedback fusion module, and the green block represents the attention module.

Figure 1 shows the skin lesion segmentation network architecture FAC-Net proposed based on CE-Net. Specifically, the FAC-Net network makes the CE-Net network as the backbone architecture. Then, we used the FFB in the down-sampling stage, which enables the network to get richer feature map information during the down-sampling process. The AMB attention module is used in the up-sampling stage to get critical information from the numerous information of the feature map. Compared with the previously proposed Attention Gate (AG) [44] module that directly uses the global information at the skip connection to generate the attention weight map. Finally, the generated weight plot was used to weight the output of each up-sampling layer. The special feature of the proposed network is that the idea of feedback fusion is used in the information-rich down-sampling layer and the idea of attention module weighting is used in the up-sampling recovery of the feature map, and the two ideas are further combined to achieve the reuse of the feature map and the emphasis of the key information.

### 3.2. Feedback Fusion Block

In the deep learning framework, encoding and decoding structures are usually used to extract map features. The down-sampling part comprises convolution and pooling modules. Although in the process of convolution and pooling operations, the network will continue to extract the feature information of the input image. Still, in the process of extraction, the network will also lose or ignore some critical feature information. Due to the considerable complexity of skin lesions, it is particularly vital to get global information when dealing with the collection of images of skin lesions. Therefore, how to reduce the loss of critical information has become an urgent problem for us to solve. Aiming at this practical problem, this paper presents a solution. The feedback mechanism is able to reuse the parameter information and get richer feature information. Considering that this paper is a research discussion without doing data enhancement, it is extremely important to reuse the existing feature information of datasets in the network to obtain information that is comparable or even richer than after data enhancement. For this reason, this paper presents a novel and efficient FFB modules guided by the feedback mechanism as a theory. The reuse of feature information also occurs in previous works, such as feature pyramid network (FPN) [45], which employs a top-down network structure with lateral connections to make predictions on each layer of feature maps. The output feature maps from each layer are up-sampled by the output feature maps from the previous layer and summed from the feedforward feature maps whose size is consistent with that feature map. While FFB presented in this paper is different from FPN. FFB module, instead of the previous direct feedback way, the upper-level feature information map is fed back to the lower-level in the form of feature weight matrix by top-down feedback, and along the top-down feedback direction, respectively, to obtain the output of each layer feature map after feedback fusion. Specifically, feature information obtained from two adjacent down-sampling layers is added to the FFB module and then merged with corresponding up-sampling layers through skip connection. Such a processing way obtains feature information that is more relevant to the skin lesions without changing the extraction operation. At the same time, we have proven through experiments that the FFB module can effectively improve the segmentation effect of skin lesions.

**Figure 2:**
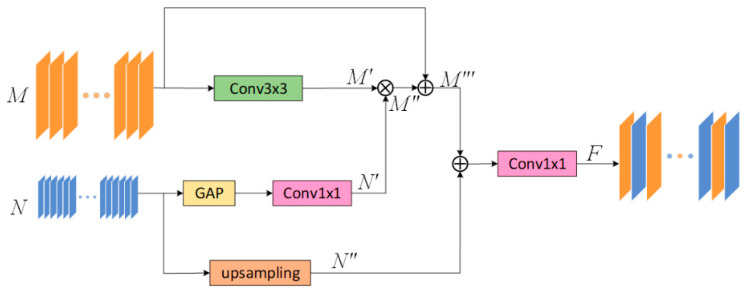
The design of the feedback fusion module (FFB). It improves the ability of feature reuse by performing feedback fusion on two adjacent layers.

Specifically, FFB consists of two parts of the input. Take the FFB module between the two coding layers E1 and E2 as an example. As shown in Figure 2, we use the E1 encoding output feature map $M\in R^{C\times H\times W}$ as the input of the first part of this module. The operation flow of FFB operation is shown in Table 1.

In the first part of this module, given a feature map M, after the convolution operation, the feature is extracted without changing the channel and resolution to obtain M′. The above-mentioned extracted critical feature information $M\prime \in R^{C\times H\times W}$ is multiplied with the feature weight map $N\prime \in R^{C\times 1\times 1}$ input from the second part by element to get the enhanced feature map $M\prime \prime \in R^{C\times H\times W}$. The weighted feature map M′′ is spliced with the original feature map M to obtain the fused output feature map $M\prime \prime \prime \in R^{2C\times H\times W}$ of the first part.

In the second part of this module, Given the feature map $N\in R^{2C\times H/2\times W/2}$ output by E2 after passing through the encoding part of the FFB module is used as the input. The feature map N will extract the maximum value of each channel through maximum global pooling, then through a 1 × 1 convolution operation, a maximum weight value map $N\prime \in R^{C\times 1\times 1}$. In addition, we will directly pass the original input feature map N to up-sampling, in this way, a new feature map $N\prime \prime \in R^{2C\times H\times W}$ is obtained. Finally, the feature map M′′′ and the feature map N′′ are spliced together, then the number of channels is restored through a 1 × 1 convolution and the output feature map $F\in R^{C\times H\times W}$ is obtained.

Through the operations described above, we will continuously strengthen the critical information of the input images. In the end, a map with enhanced feature information is used as the input part of the skip connection, and it is input into an up-sampling layer with the same resolution as the feature map.

### 3.3. Attention Mechanism Block

**Figure 3:**
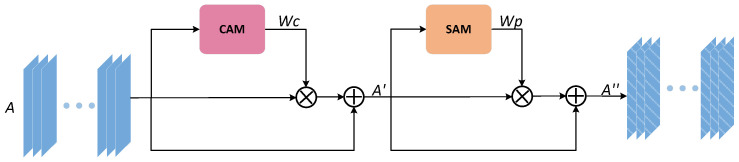
The improved architecture of the attention mechanism block (AMB) module.

In this paper, feedback fusion modules do have a good impact on the reusability of model parameters, reducing a large number of parameters, but as such structures all transfer feature information in the form of iterative rounds during training, redundant feature information as well as some noise may have an additive effect in iterations and affect the convergence of the network as well as the final effect. To suppress the side effects produced based on FFB modules, this paper improved the CBAM attention module and named it the AMB module, whose structure is identical to that of the CBAM module (Channel attention mechanism (CAM) and Spatial attention mechanism (SAM) are connected in series). At the same time, given the large degree of similarity between feature maps of multi-channels taken in the network architecture, the most recurrent feature information occurring in each channel on a spatial scale is vital feature information (that is, what we need to acquire). Therefore, this paper proposes the addition of an algorithmic branch that solves mode values in Sam modules to enforce the ability of Sam to screen important feature information. The structure of AMB as shown in Figure 3, the up-sampling feature map $A\in R^{C\times H\times W}$ after skip connection splicing is sent to the AMB module as input. First, we obtain a weight map $W_{C}\in R^{C\times 1\times 1}$ and a feature map $A\prime \in R^{C\times H\times W}$ sequentially through CAM. Then, the A′ is used as the input of the SAM to obtain the spatial weighted image $W_{P}\in R^{C\times 1\times 1}$. In the same way, $W_{P}$ and A′ are multiplied by elements and then spliced with the initial input A′ to get the final output map $A\prime \prime \in R^{C\times H\times W}$.

#### 3.3.1. Channel Attention Mechanism

**Figure 4:**
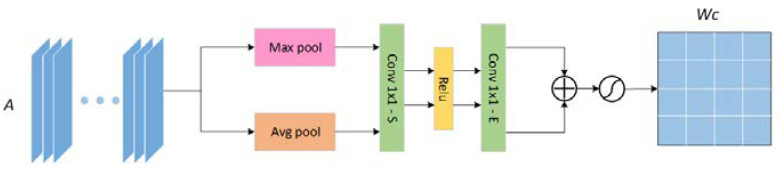
The architecture of the channel attention mechanism (CAM) module.

The channel attention uses the traditional compression and expansion attention mechanism. As observed from Figure 4, the input feature map A is sequentially global pooling, compression, activation, expansion, and normalization to get the channel weight map $W_{C}$, to prepare for the subsequent weighting of the feature map.

#### 3.3.2. Spatial Attention Mechanism

For the spatial attention mechanism, we have made corresponding improvements. We consider that the maximum value and average value obtained in the corresponding spatial position of each pixel in the feature map are the key features of the current situation in the spatial channel. Still, some positions in the space may have extreme values. In response to this phenomenon, we propose to calculate the mode value of the same position in the space and obtain a mode value feature weight map. In the network, the image changes the size of the feature map and the number of channels through convolution and pooling, which continuously obtains vital information. Therefore, we believe that the features acquired at each position of the feature map in the corresponding position in the space will have similarities. The feature that appears the most times is the key information.

**Figure 5:**
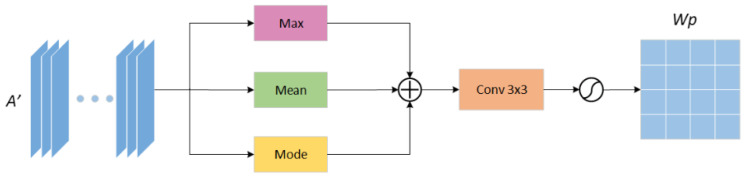
Improved channel attention mechanism (CAM) module architecture to obtain. Spatial attention weight map.

In this paper, by calculating the features that appear the most in their corresponding spatial location, we get the mode value weight map. The previous maximum feature weight map and average feature weight map are supplemented to obtain the maximum feature weight map, average weight map, and mode value feature weight map. The three feature maps are element-wise added to effectively weigh various features, suppress invalid information, and amplify vital information, to improve the accuracy of segmenting skin lesion feature maps. As shown in Figure 5, the input feature map is the feature map $A\prime \in R^{C\times H\times W}$ enhanced by channel attention, and the maximum value, average value, and mode value are extracted, respectively. After the channel is merged, the spatial weight map $W_{P}\in R^{C\times 1\times 1}$ is normalized by Softmax function.

### 3.4. Loss Function

In order to optimize the training of the network, the loss function is suggested to detect the error between the prediction result of the network output and the GT image to continuously reduce the difference between the two during training. The smaller the value, the better our results. In order to optimize our model and make the model converge quickly and stably during the training process, we have selected the most classical dice loss function-Dice Loss. The Dice coefficient originates from two classification tasks, and is generally used as an index to evaluate the degree of overlap between two samples. The index ranges from 0 to 1, where “1” means complete overlap. As shown in the Equation (1), Diss loss is one minus the Dice coefficient, so the smaller the Dice Loss value, the better. Where i is the index of each pixel on the feature map, yi is the *i*th element on the GT, and pi is the *i*th element of the network prediction SR.

(1) $$L_{DICE}=1-2\frac{\sum_{i}y_{i}p_{i}}{\sum_{i}y_{i}+\sum_{i}p_{i}}$$

## 4. Experiment

In this part, we first explain the selected datasets and evaluation indicators. Then, we introduce the detailed parameter settings of the training process. Next, we conduct ablation experiments on various modules. Last, we show the results of FAC-Net.

### 4.1. Datasets

**Figure 6:**
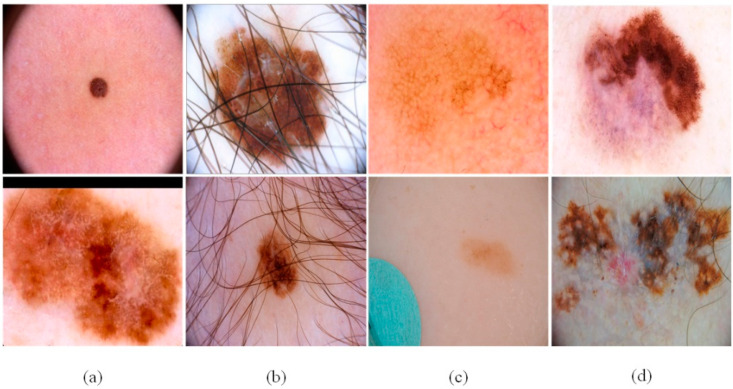
The challenges of skin lesion segmentation: (**a**) irregular lesion area, (**b**) case with fair and air bladder in lesions, (**c**) small changes between normal skin and skin lesion, and (**d**) the stratified features of the lesions.

We used three accepted skin image datasets (ISBI2016 [46], ISBI2017 [47], and ISIC2018 [48]), to verify the network proposed in this article. There are several types of skin lesions in the datasets, as shown in Figure 6:

- The size and shape of the skin lesions in the sample are different, and the boundary is fuzzy.
- There are interfering factors in the sample, such as hair, air bubbles and other obstructions.
- The distinction between the diseased part and the normal skin part is small and difficult to distinguish.
- There are obvious hierarchical features in the lesion location of the sample, which may lead to misjudgment of the lesion boundary.

These problems make the task of segmenting skin lesions extremely difficult. Considering the actual clinical medical environment, we did not perform any processing on the data. We only adjusted all the images and GT to a resolution of 256 × 256.

The 2594 images with GT were provided in the ISIC2018 dataset as the training dataset. Provide about 100 images without GT for the verification dataset and 1000 images without GT for the test dataset. However, since the dataset does not provide the GT maps corresponding to the images in the validation and test dataset, we divide the training dataset into three parts at a ratio of 7:1:2, which are respectively used as the training dataset, the validation dataset, and test dataset.

Training images of the ISBI2017 dataset includes 2000 dermoscopy images of different resolutions and the corresponding segmentation GT maps. The test images consist of 600 dermoscopy images and corresponding segmentation label maps. For this dataset, we select the original training images as the training dataset. At the same time, we divide the test images at a ratio of 1:4 as the verification dataset and the test dataset.

ISBI2016 dataset contains 900 training images in JPEG format and 379 test images. These images are classified and annotated by clinical experts and then encoded as single-channel segmentation label images. For the ISBI2016 dataset, the training scheme adopted is: the 900 training images provided by ISBI2016 dataset as the training dataset, and the test images are split into a verification dataset and a test dataset at a ratio of 1:4.

### 4.2. Metrics

To quantitatively evaluate the segmentation ability of the feedback attention network FAC-Net, we use the following widely recognized segmentation evaluation indicators. Sensitivity (SE) Equation (2) represents the proportion of skin lesion pixels that are correctly segmented. The higher the sensitivity, the closer to 1.0, and the closer to 1.0, the better the segmentation effect. Specificity (SP) Equation (3) represents the proportion of pixels that are not correctly segmented in the undamaged skin part. Higher specificity means that as many negative instances as possible are judged as undesirable. The normal skin area is considered being normal, and there is no misjudgment. Precision (PC) Equation (4) is also used as an evaluation indicator. In addition, the Jaccard index (JA) Equation (5) and Dice coefficient (DC) Equation (6) are used to measure the similarity between the segmentation result and the marked GT. Accuracy (ACC) Equation (7) is also used to display the overall pixel-level segmentation performance, and the formula is shown below.

(2) $$\;\mathrm{SE}=\frac{\mathrm{TP}}{\mathrm{TP}+\mathrm{FN}}$$

(3) $$\mathrm{SP}=\frac{\mathrm{TN}}{\mathrm{TN}+\mathrm{FP}}$$

(4) $$\;\mathrm{PC}=\frac{\mathrm{TP}}{\mathrm{TP}+\mathrm{FP}}$$

(5) $$\mathrm{JA}=\frac{\mathrm{TP}}{\mathrm{TP}+\mathrm{FN}+\mathrm{FP}}$$

(6) $$\mathrm{DC}=\frac{2\;\times \;\mathrm{TP}}{2\;\times \;\mathrm{TP}+\mathrm{FP}+\mathrm{FN}}$$

(7) $$\mathrm{ACC}=\frac{\mathrm{TN}+\mathrm{TP}}{\mathrm{TP}+\mathrm{TN}+\mathrm{FP}+\mathrm{FN}}$$

Among them, in the skin lesion area, TP represents pixels that are correctly segmented, and FN represents pixels that are not correctly segmented. On the contrary, in a normal skin area, TN represents normal pixels are correctly segmented, and FP represents normal pixels are not correctly segmented. Through the above indicators, we can objectively evaluate the accuracy of segmentation.

### 4.3. Experimental Setting

Firstly, we apply the well-known Kaiming initialization method to optimize the training of the network. Then use of ADAM optimizer to perform optimization in this article. Next, the learning rate, the batch size and the epoch are set to 0.0001, 12, and 200, respectively. In our experiment our model can converge stably after 200 training cycles.

### 4.4. Ablation Experiment

**Figure 7:**
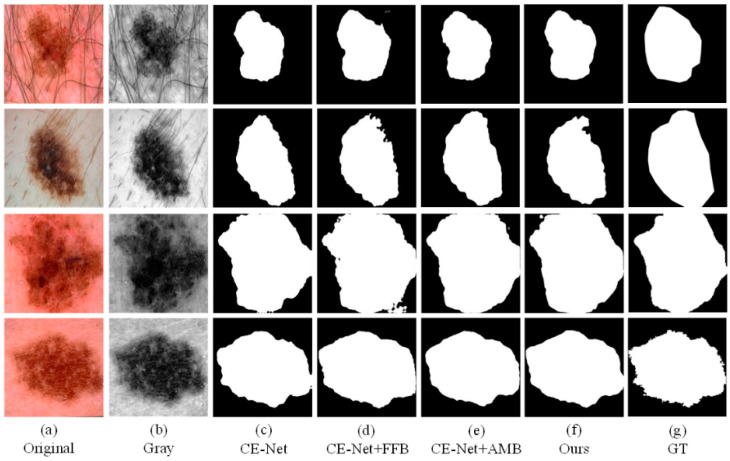
A visualized comparison chart of each network in the ablation experiment. (**a**,**b**) represent the original image and gray-scale image input into the network compared with (**c**,**d**). After adding the FFB module, the information obtained by the network is more abundant, and slightly larger than that of the GT diagram. Comparing with (**c**,**e**), it can be obtained that adding the AMB module can suppress irrelevant information. Comparing (**c**–**g**), it can be concluded that the network with two modules added at the same time has the best segmentation result.

To better demonstrate the effects that different modules proposed in this article. We conducted ablation experiments. In the ablation experiment, we test CE-Net, CE-Net+FFB, CE-Net+AMB, and the method proposed in this article (CE-Net+FFB+AMB) on the ISIC2018 dataset. We compare the effectiveness of the separate modules on the effect of segmenting the lesion. As shown in Figure 7, we can observe that the original CE-Net cannot obtain satisfactory segmentation results, especially when the lesions have different locations and shapes (Figure 6a). Compared with CE-Net, the CE-Net+FFB method will obtain richer features after adding the FFB module. Even if the skin lesion has irregular shapes and fuzzy edges, the FFB feedback module can fully capture the lesion area. The segmentation result is preferable to CE-Net. FFB module itself obtains the part of the diseased part through continuous supplementation and fusion. Therefore, when the contour of the lesion is irregular or fuzzy, the lesion area obtained by the FFB feedback network will be slightly larger than the true value of the label. Contrary to this situation, we can see that CE-Net+AMB can effectively remove some irrelevant information, to segment the lesion into segmented regions closer to the GT. Therefore, face with the complex datasets mentioned above, we take the advantages of the two modules as the starting point and propose an algorithm framework of CE-Net+FFB+AMB (FAC-Net) to deal with the challenges brought about by this complexity. The segmentation performance of the skin lesions of each ablation network is clearly demonstrated in the experiment: the segmentation results of the CE-Net+FFB+AMB are better than CE-Net, CE-Net+FFB, and CE-Net+AMB. The segmentation comparison results are shown in Figure 7. The segmentation maps of results can effectively prove the effectiveness and accurateness of the FAC-Net method proposed in this paper.

In addition, we also performed statistics and comparisons on the JA, DC, SE, PC, SP, and ACC values of different methods. As shown in Table 2, we can see clearly that the segmentation results of CE-Net+FFB and CE-Net+AMB are better than the traditional CE-Net, which also proves the effectiveness of the FFB and AMB modules proposed in this paper. In addition, the above evaluation indicators can confirm that in these four experiments, CE-Net+FFB+AMB can achieve an excellent skin lesion segmentation effect.

To further verify the effectiveness of the spatial attention mechanism in the AMB module, we conducted the following verification experiments for the spatial attention mechanism, and obtained statistical values such as JA, DC, SE, SP, and ACC. The verification experiments we carried out are shown in Table 3, in which Mode stands for the mode function, Max stands for the maximum value function, and Avg stands for the average value function. It can be seen from the statistical table that the AMB method proposed in this paper can obtain the best training effect in all function algorithm combinations.

### 4.5. Comparative Experiment

We also conducted comparative experiments between the proposed segmentation network and the mature segmentation network, including U-Net, R2U-Net, CE-Net, SA-UNet, and UNet3+. In order to ensure the fairness of the experiment comparison, we conduct experiments under the same parameter settings and computing environment. We apply each network to three datasets (ISBI2016, ISBI2017, ISIC2018). Binary images of skin lesions obtained by network training segmentation are shown in the three figures below.

**Figure 8:**
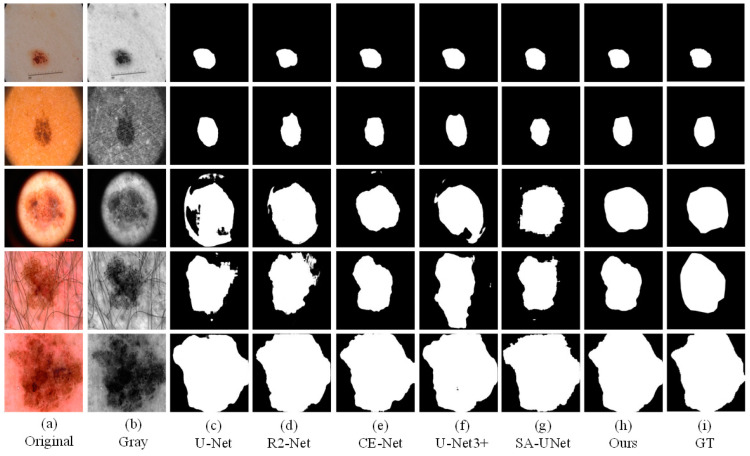
The visual segmentation comparison chart of each comparison network on ISIC2018. (**a**,**b**) represents original and gray-scale images of input, (**i**) represents GT maps. (**c**–**g**) represent the segmentation results of comparison networks, respectively. (**h**) represents the segmentation result of our proposed method, from which it can be seen that the segmentation result of our proposed method is the closest to the GT maps.

**Figure 9:**
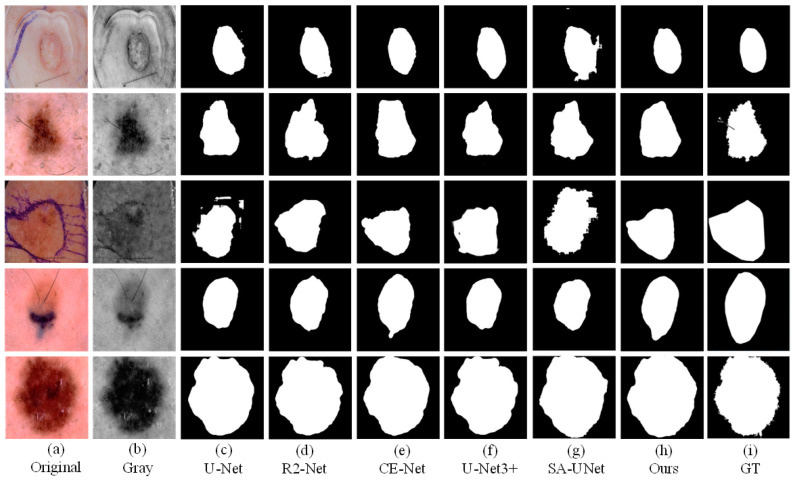
The visual segmentation comparison chart of each comparison network on ISIC2017. (**a**,**b**) represents original and gray-scale images of input, (**i**) represents GT maps. (**c**–**g**) represent the segmentation results of comparison networks, respectively. (**h**) represents the segmentation result of our proposed method, from which it can be seen that the segmentation result of our proposed method is the closest to the GT maps.

**Figure 10:**
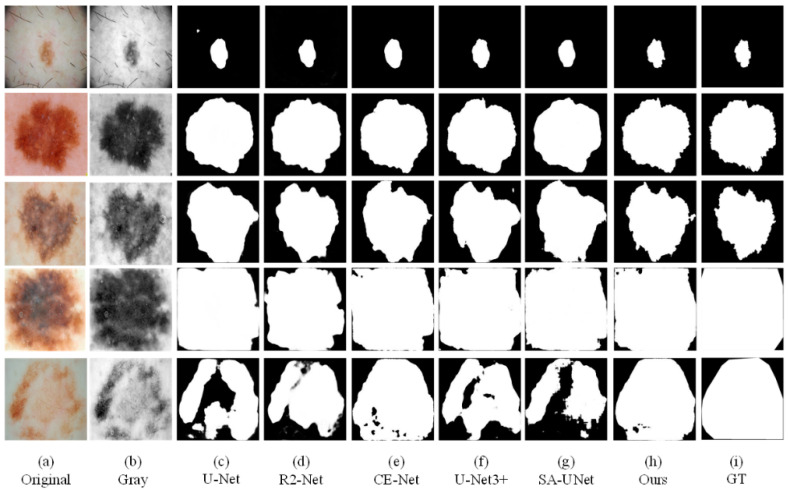
The visual segmentation comparison chart of each comparison network on ISBI2016. (**a**,**b**) represent original and gray-scale images of input, (**i**) represents GT maps. (**c**–**g**) represent the segmentation results of comparison networks, respectively. (**h**) represents the segmentation result of our proposed method, from which it can be seen that the segmentation result of our proposed method is the closest to the GT maps.

As the figures (Figure 8, Figure 9 and Figure 10) show, we can see that U-Net usually cannot accurately identify the complex boundaries of challenging cases. Performance and segmentation results of R2U-Net based on recursive residual convolution block are better than U-Net. CE-Net achieves higher segmentation accuracy by combining dense dilated convolution module and residual multi-core pool. SA-UNet introduces a spatial attention module and performs adaptive feature refinement and obtains excellent results in retinal segmentation. However, the segmentation results obtained as a result of this network on the skin disease datasets are only better than U-Net. U-Net3+ further optimizes the segmentation results of skin lesions by using full-scale skip connection and deep supervision in the network, but it takes up a lot of memory. The experiment results show that CE-Net+FFB+AMB have obtained segmentation maps that are preferable than other methods on the whole, and achieved higher skin lesion segmentation accuracy.

Besides intuitive comparison, we also statistically compared the data (ACC, SE, SP, PC, JA, and DC) obtained from the experiments on three datasets (ISBI2016, ISBI2017, ISIC2018). In the three tables (Table 4, Table 5 and Table 6) below, it can be seen that the method we propose is superior to the comparison networks in most indicators. Compared with the primary network CE-Net, the approach proposed in the present paper has a significant improvement in the three datasets. It is worthwhile to note that on the 2017 dataset, the method proposed in this article compared with the basic network also have corresponding improvements in various indicators. Also on the 2016 dataset, the method proposed in this article is greater than competitors in terms of ACC, SP, PC, JA, and DC. Through the comparison of the values of the indicators for ACC, PC, JA, and DC on the three datasets (ISIC2018, ISBI2017, ISBI2016), our proposed module is shown to be robust in improving the segmentation results of skin lesions.

In order to better verify the effectiveness of the proposed method, we directly compare it with State-of-the-Art Methods. In the absence of data enhancement, the network we proposed achieves better results compared with other networks that use data enhancement for segmentation. As shown in Table 7, the method we proposed has corresponding improvement compared with other networks.

## 5. Discussion and Conclusions

Through the above-mentioned ablation research and comparative experiments, we found that although segmentation of skin lesions has an immense challenge, after integrating the two modules of FFB and AMB, our method achieved better results. However, at present, our method still has room for optimization. Similar to most CNN networks, facing the challenge of too minor differences between normal skin and skin lesions, our approach may not get an accurate segmentation boundary, but compared to other contrast networks, our segmentation results are still the closest to the GT image.

In summary, without data augmentation, a novel and efficient network model for skin lesion segmentation is proposed and implemented in this paper, which is called FAC-net. We introduce the idea of feedback fusion combined with attentional mechanisms. Specifically, in the coding section, in order to effectively feedback the high-level output information to the low-level output to adjust its output and obtain richer feature information, we designed a novel FFB and applied it between adjacent coding layers. In the decoding section, to better carry out the screening of focused information, highlight the target information, and reduce the background information, we adopted AMB and embedded it after the information fusion of skipping connection. The main advantage of our network over other existing networks is the ability to also get rich and focused information in the absence of data augmentation. To verify the validity of the network proposed in this paper, we evaluate it using three publicly available datasets (ISIC2018, ISBI2017, ISBI2016). Through plenty of ablation experiments, we respectively verified the feasibility and efficacy of the two blocks, and also demonstrated that the two could achieve the best results in the case of combination. Through extensive contrast experiments, the effectiveness of the network presented in this paper in skin lesion segmentation tasks without doing data enhancement is well demonstrated.

In future work, we will conduct research improvements on the network proposed in this paper in the following three aspects. First, we will investigate in the model the operation of improving the feedback fusion mechanism to transform the high-level information into feature weight matrix maps, so that the upper-level information features can better feedback into the lower-level feature maps. Second, for the targeted acquisition of skin lesion location in feature maps, we will explore new attentional mechanism modules that are more suitable for skin lesions, optimizing the placement and number of such modules. Third, we will look for loss functions that can further narrow the difference between training results and label values during the training process, such that the skin lesion segmentation effect is further boosted.

## Figures and Tables

**Table 1:** Operation of FFB module.

Process	Operation	Input	Output
Part one	Conv3 × 3	C × H × W	C × H × W
	element-wise Multi	C × H × W & C × 1 × 1	C × H × W
	Concat	C × H × W & C × H × W	2C × H × W
Part two	Gap	2C × H/2 × W/2	2C × 1 × 1
	Conv1 × 1	2C × 1 × 1	C × 1 × 1
	Up-sampling	2C × H/2 × W/2	2C × H × W
Connection	Concat	2C × H × W & 2C × H × W	4C × H × W
	Conv1 × 1	4C × H × W	C × H × W

**Table 2:** Evaluation index of each network in ablation experiment.

Model	ACC (%)	SE (%)	SP (%)	PC (%)	JA (%)	DC (%)
CE-Net	95.81	88.11	97.88	91.64	81.58	89.71
CE-Net+FFB	96.18	88.40	98.21	92.00	82.83	90.49
CE-Net+AMB	96.05	89.74	97.75	90.49	82.87	90.48
CE-Net+FFB+AMB	96.41	89.92	98.16	92.74	83.99	91.19

**Table 3:** Ablation experiment on AMB module.

Model	ACC (%)	SE (%)	SP (%)	PC (%)	JA (%)	DC (%)
Mode	95.96	90.17	97.47	90.63	82.64	90.24
Mode+Avg	96.10	89.62	97.92	91.86	82.94	90.55
Mode+Max	96.07	89.54	97.82	91.64	82.79	90.45
Max+Avg	96.17	90.02	97.64	91.23	82.83	90.45
Mode+Max+Avg	96.41	89.92	98.16	92.74	83.99	91.19

**Table 4:** Evaluation indicators of each comparative network on ISBI2018.

Model	Year	ACC (%)	SE (%)	SP (%)	PC (%)	JA (%)	DC (%)
U-Net	2015	94.66	86.03	97.10	88.72	77.43	87.13
R2U-Net	2018	95.09	86.58	97.51	90.00	78.85	88.05
CE-Net	2019	95.81	88.11	97.88	91.64	81.58	89.71
U-Net3+	2020	94.97	85.20	97.77	90.86	78.30	87.71
SA-UNet	2021	94.78	84.87	97.59	90.29	77.63	87.25
Ours	-	**96.41**	**89.92**	**98.16**	**92.74**	**83.99**	**91.19**

**Table 5:** Evaluation indicators of each comparative network on ISBI2017.

Model	Year	ACC (%)	SE (%)	SP (%)	PC (%)	JA (%)	DC (%)
U-Net	2015	92.21	74.38	97.58	89.58	68.30	80.70
R2U-Net	2018	92.28	75.37	97.45	89.38	69.04	88.05
CE-Net	2019	93.49	80.51	97.33	89.92	73.83	84.55
U-Net3+	2020	92.08	72.95	**97.87**	90.69	67.79	80.29
SA-UNet	2021	92.08	76.93	96.66	86.74	68.76	81.06
Ours	-	**93.63**	**81.06**	97.43	**90.07**	**74.27**	**84.91**

**Table 6:** Evaluation indicators of each comparative network on ISBI2016.

**Model**	**Year**	**ACC (%)**	**SE (%)**	**SP (%)**	**PC (%)**	**JA (%)**	**DC (%)**
U-Net	2015	94.69	91.30	96.01	89.32	82.18	90.12
R2U-Net	2018	94.43	87.68	97.06	91.49	80.95	89.38
CE-Net	2019	95.94	**92.80**	97.10	92.06	85.85	92.31
U-Net3+	2020	94.94	90.26	96.74	91.12	82.87	90.54
SA-UNet	2021	94.11	89.46	95.90	88.82	80.14	88.82
Ours	-	**96.09**	92.50	**97.43**	**92.74**	**86.23**	**92.51**

**Table 7:** Comparisons with State-of-the-Art Methods.

Model	Dataset	ACC (%)	SP (%)	JA (%)	DC (%)
Tang et al. [32]-2020	ISBI2016	96.08	-	85.98	91.91
Hafhouf et al. [30]-2020	ISBI2016	93.9	95.2	82.7	89.6
Khan et al. [28]-2021	ISBI2016	92.69	-	-	-
Ours	ISBI2016	**96.09**	**97.43**	**86.23**	**92.51**
Tong et al. [29]-2021	ISBI2017	92.6	96.5	74.2	83
Ours	ISBI2017	**93.63**	**97.43**	**74.27**	**84.91**
Salih et al. [27]-2020	ISIC2018	89.47	95.09	72.45	80.67
Tang et al. [32]-2020	ISIC2018	-	-	81.91	-
Saha et al. [31]-2020	ISIC2018	-	93.2	81.9	89.1
Wu et al. [33]-2021	ISIC2018	94.7	94.1	84.4	90.8
Khan et al. [28]-2021	ISIC2018	92.69	-	-	-
Ours	ISIC2018	**96.41**	**98.16**	**83.99**	**91.19**

## Data Availability

We used two classical dermoscopy datasets to evaluate the proposed segmentation network. They are respectively ISIC2018 dataset, ISBI2017 dataset and ISBI2016 dataset. The url of these datasets is https://challenge.isic-archive.com/data (accessed on 27 July 2021).
